# Genomic Profiling Identifies *GATA6* as a Candidate Oncogene Amplified in Pancreatobiliary Cancer

**DOI:** 10.1371/journal.pgen.1000081

**Published:** 2008-05-23

**Authors:** Kevin A. Kwei, Murali D. Bashyam, Jessica Kao, Raman Ratheesh, Edumakanti C. Reddy, Young H. Kim, Kelli Montgomery, Craig P. Giacomini, Yoon-La Choi, Sreejata Chatterjee, Collins A. Karikari, Keyan Salari, Pei Wang, Tina Hernandez-Boussard, Gowrishankar Swarnalata, Matt van de Rijn, Anirban Maitra, Jonathan R. Pollack

**Affiliations:** 1 Department of Pathology, Stanford University, Stanford, California, United States of America; 2 Laboratory of Molecular Oncology, Centre for DNA Fingerprinting and Diagnostics, Nacharam, Hyderabad, India; 3 National Genomics and Transcriptomics Facility, Centre for DNA Fingerprinting and Diagnostics, Nacharam, Hyderabad, India; 4 Department of Pathology, Samsung Medical Center, Sungkyunkwan University School of Medicine, Seoul, South Korea; 5 Department of Pathology, The Johns Hopkins University, Baltimore, Maryland, United States of America; 6 Department of Genetics, Stanford University, Stanford, California, United States of America; 7 Division of Public Health Sciences, Fred Hutchinson Cancer Research Center, Seattle, Washington, United States of America; 8 Department of Pathology, Apollo Hospitals, Hyderabad, India; The Jackson Laboratory, United States of America

## Abstract

Pancreatobiliary cancers have among the highest mortality rates of any cancer type. Discovering the full spectrum of molecular genetic alterations may suggest new avenues for therapy. To catalogue genomic alterations, we carried out array-based genomic profiling of 31 exocrine pancreatic cancers and 6 distal bile duct cancers, expanded as xenografts to enrich the tumor cell fraction. We identified numerous focal DNA amplifications and deletions, including in 19% of pancreatobiliary cases gain at cytoband 18q11.2, a locus uncommonly amplified in other tumor types. The smallest shared amplification at 18q11.2 included *GATA6*, a transcriptional regulator previously linked to normal pancreas development. When amplified, *GATA6* was overexpressed at both the mRNA and protein levels, and strong immunostaining was observed in 25 of 54 (46%) primary pancreatic cancers compared to 0 of 33 normal pancreas specimens surveyed. *GATA6* expression in xenografts was associated with specific microarray gene-expression patterns, enriched for GATA binding sites and mitochondrial oxidative phosphorylation activity. siRNA mediated knockdown of *GATA6* in pancreatic cancer cell lines with amplification led to reduced cell proliferation, cell cycle progression, and colony formation. Our findings indicate that *GATA6* amplification and overexpression contribute to the oncogenic phenotypes of pancreatic cancer cells, and identify *GATA6* as a candidate lineage-specific oncogene in pancreatobiliary cancer, with implications for novel treatment strategies.

## Author Summary

Pancreatic cancer is a devastating disease, having among the lowest survival rates of any cancer. A better understanding of the molecular basis of pancreatic cancer may lead to improved rationale therapies. We report here the discovery of amplification (i.e. extra copies) of the *GATA6* gene in many human pancreatic cancers. GATA6 is a regulator of gene expression and functions in the development of the normal pancreas. Our findings indicate that its amplification and aberrant overexpression contribute to pancreatic cancer development. GATA6 joins a growing list of cancer genes with key roles in normal human development but pathogenic roles in cancer when aberrantly expressed. Our discovery of GATA6 amplification provides a new foothold into understanding the pathogenic mechanisms underlying pancreatic cancer, and suggests new strategies for therapy by targeting GATA6 or the genes it regulates.

## Introduction

Pancreatic cancer has among the highest mortality rates of any cancer, pointing to a critical need for more effective therapies. While much progress has been made in understanding pancreatic cancer pathogenesis, a more comprehensive characterization of molecular genetic alterations is needed to define new molecular targets and therapeutic opportunities [1].

Genomic DNA copy number alterations (CNAs) are frequent in pancreatic cancer, where they alter the dosage and expression of cancer genes. Amplified oncogenes include *KRAS* (also commonly activated by point mutation), *AKT2* and *MYB*. Likewise, deleted tumor suppressor genes (TSGs) include *CDKN2A*, *TP53* and *SMAD4* (also inactivated by mutation and promoter hypermethylation) [2]. Mapping CNAs has become an important starting point for discovering new cancer genes, and indeed led to the original identification of *CDKN2A* and *SMAD4* as TSGs [3],[4].

During development, the ventral portion of the pancreas arises from the primitive bile duct [5]. While less is known of extrahepatic bile duct cancers, they appear to share many features with pancreatic cancers, including frequent molecular alterations of *KRAS*, *CDKN2A*, *TP53* and *SMAD4*, as well as global patterns of allelic loss [6],[7]. Because of their anatomic proximity and similar histologies, pancreatic and distal bile duct cancers can at times be difficult to distinguish, and from a clinical and research standpoint are often practically combined under the umbrella of pancreatobiliary cancer.

Recently, array-based comparative genomic hybridization (array CGH) has provided a powerful approach to catalog CNAs in cancer genomes [8],[9]. Profiling pancreatobiliary cancers, however, presents unique technical challenges due to the strong stromal response, where tumor cells typically account for less than 20% of cells in the specimen. Not surprisingly, to date genomic profiling of pancreatic cancer has been largely confined to studies of derived cell lines [10]–[13]. Strategies to enrich for tumor cells from primary cancers include physical microdissection, which is both technically demanding and, with the low yield of genomic DNA, is subject to biases in subsequent target amplification. An alternative strategy is expanding tumors as xenografts in nude mice, which effectively enriches the tumor cell fraction to >95%, (with the remaining stromal cells being of murine origin), while preserving genomic alterations of the parental tumor [14]. Here, we apply genomic profiling to a set of pancreatic and distal bile duct cancers grown as xenografts in nude mice, where among other alterations we identify and characterize *GATA6* as a novel candidate lineage-specific oncogene amplified in pancreatobiliary cancer.

## Results

To comprehensively catalog CNAs in pancreatobiliary cancers, we carried out array CGH-based genomic profiling of a set of 37 cancers (31 exocrine pancreatic cancers and 6 distal bile duct cancers) expanded as xenografts to enrich for tumor cells, using cDNA microarrays representing ∼22,000 genes with a median interprobe spacing of ∼15 Kb. We identified numerous CNAs, among which 17 focal high-level DNA amplifications (i.e. fluorescence ratios ≥3, corresponding to at least 5-fold amplification [8]) and 7 presumptive homozygous deletions (i.e. fluorescence ratios ≤0.25) were particularly informative in pinpointing known or novel candidate cancer genes (Table 1). By profiling gene expression in parallel, we also defined the subset of amplified genes exhibiting elevated expression (Table 1), a characteristic of oncogenes. For a subset of presumptive homozygous deletions, we validated homozygous loss by polymerase chain reaction (PCR) using human gene-specific primers (Table 1 and Figure S1).

**Table 1:** High-level amplifications and homozygous deletions.

Cytoband	P-Border (Kb)	Q-Border (Kb)	Size (Kb)	Specimen^a^	Frequency CNA (%)^b^	Selected resident genes^c^^,^ d
**Amplifications**						
4q12	57,213	57,681	468	B291	3	***REST***, *IGFBP7*
6p21.2	39,162	39,406	244	P410	3	*KCNK5*
7q22.1	97,489	100,136	2647	P178	19	***TRRAP*** **, ** ***SMURF1*** **, ** ***MCM7***
8q24.21	128,135	130,155	2020	P167	54	*MYC*, ***PVT1***
9p13.3	34,369	34,656	287	P374	27	*CNTFR*
11p11.2	45,885	46,363	478	P154, P155	22	*CREB3L1*, ***MDK***
12p12.1	24,854	27,498	2644^e^	P354, B291	22	***KRAS***
12q15	68,070	69,445	1374	B291	8	***YEATS4***, ***FRS2***, ***PTPRR***
17q21.2	36,755	36,925	170	P374	27	***KRT15***
17q21.31	39,624	39,993	369	P294	27	***SLC25A39***, *IGTA2B*
17q21.33	46,183	46,513	329	P294	27	***CROP*** **, ** ***TOB1***
18q11.2	18,001	18,287	286	B291	19	***GATA6***
19p13.3	2,182	2,279	97	P165	27	*DOT1L*, *OAZ1*
19p13.2	7,609	7,654	45	P169	11	***TRAPPC5***
19q13.2	43,769	45,520	1751	B265	30	***PAK4***, ***AKT2***
19q13.3	48,364	48,927	564	P155	11	*PLAUR*
20q13.12	43,472	43,789	316	P226	19	***PIGT***
**Deletions**						
3p24.1	30,025	30,711	685	P224	16	*TGFBR2*
4q35.1	187,380	187,509	129	B301	41	*TLR3*
5q23.1	115,701	115,755	54	P266	27	
8p23.1	6,781	7,759	978	P266	41	*DEFA1*
9p21.3	21,321	21,961	640^e^	P198, P291	59^f^	*CDKN2A*
9p21.2	27,219	27,521	302^e^	P201, P374	57	*IFT74*, *TEK*, *MOBKL2B*
19q13.43	63,219	63,330	111	P374	14	*ZNF135*

a Specimen(s) with high-level amplification or presumptive homozygous deletion.

b Includes low-level respective gain/loss at the same locus.

c Boldface indicates gene expression well-measured by microarray and elevated when amplified.

d Underlined genes are those confirmed homozygously deleted by PCR.

e Boundaries vary among specimens; minimum shared region indicated.

f 43% of specimens exhibited homozygous deletion by PCR.

**Figure 1:**
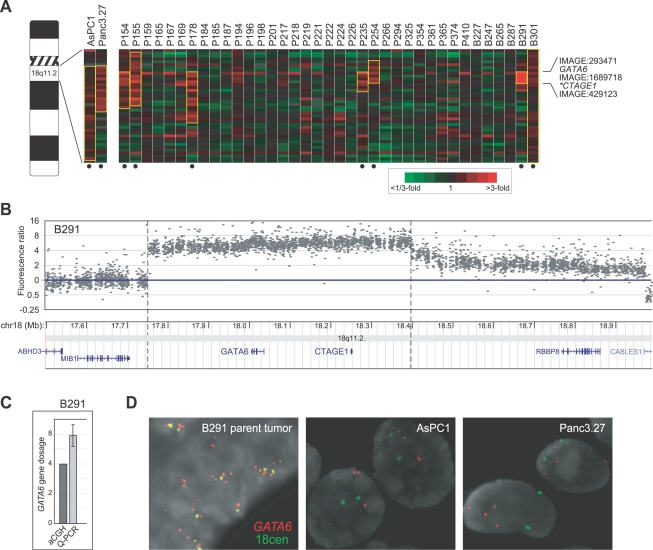
*GATA6* is focally amplified in pancreatobiliary cancer. (A) Genomic profiles by CGH on cDNA microarrays of pancreatic (P) and bile duct (B) cancer xenografts across cytoband 18q11.2. Genes are ordered by genome position. Red indicates positive tumor/normal aCGH ratios (scale shown), and samples called gained at 18q11.2 are marked below by closed circle (gains highlighted in yellow). Genes and ESTs (IMAGE clone ID shown) on the microarray residing within the amplicon core are indicated. *CTAGE1* (asterisked) was not present on the array but resides where shown. (B) Genomic profile of B291 by CGH on an Agilent ultra high-definition custom microarray tiling 18q11.2, mapped onto the UCSC genome browser (http://genome.ucsc.edu) [55]. The amplicon peak spans two genes, *GATA6* and *CTAGE1*. (C) Q-PCR validation of *GATA6* amplification in B291. Note, hybridization measurements by CGH tend to underestimate true CNA ratios [8]. (D) FISH validation of *GATA6* amplification in the parent tumor (paraffin section) from which xenograft B291 was derived (*left*), and *GATA6* gain in pancreatic cancer cell lines AsPC1 (*center*) and Panc3.27 (*right*). Gain is evident by the increased ratio of *GATA6*(red)/centromere-18(green) signals. DAPI (nuclear) counterstaining is shown in grayscale.

Among the focal amplifications, we identified gain at 18q11.2 in 19% of pancreatobiliary cancers (5 of 31 pancreatic, and 2 of 6 bile duct). Notably, we found gains spanning 18q11.2 to be less common in other tumor types we had profiled on the same array platform, including cancers of the breast (3 gains in 89 (3%) tumors, 1 in 49 (2%) cell lines), prostate (0 in 64 (0%) tumors), lung (4 in 76 (5%) tumors, 4 in 52 (8%) cell lines) and colon (1 in 29 (3%) cell lines) [15]–[17] (and unpublished data), and in these other tumor types the gains when present were not focal, suggesting the putative driver oncogene within this locus may be specific to pancreatobiliary cancer. Strikingly, the smallest shared region of amplification among the xenograft specimens spanned just two annotated genes, *GATA6* (GATA binding protein 6) and *CTAGE1* (cutaneous T-cell lymphoma (CTCL)-associated antigen 1) (Figure 1A). *GATA6* [18] belongs to the GATA factor family of transcriptional regulators, whose members are expressed in distinct developmental and tissue-specific patterns and regulate cell-restricted programs of gene expression [19]. Because *GATA6* was known to regulate normal pancreas development [20],[21], we sought to explore a possible functional connection of *GATA6* gene amplification and pancreatobiliary cancer.

A single bile duct cancer xenograft specimen (B291) with focal high-level DNA amplification was particularly informative in defining amplicon boundaries. Using a custom Agilent ultra-high definition CGH array with probes tiling 18q11.2 with an average 343nt spacing, we first confirmed the amplicon boundaries in B291, finding the amplicon peak indeed spanned just *GATA6* and *CTAGE1* (Figure 1B). We also validated *GATA6* amplification in B291 by quantitative (Q)-PCR (Figure 1C), and by fluorescence *in situ* hybridization (FISH) in the parent tumor from which the B291 xenograft was derived (Figure 1D, *left* panel), the latter excluding the possibility of amplification arising during xenograft growth. Focal 18q11.2 gain was also present in 3 of 18 (17%) pancreatic cancer cell lines (AsPC1, Panc3.27 and Capan1) we had previously profiled by array CGH ([13]) (Figure 1A, and data not shown), a finding we confirmed by FISH (Figure 1D).

**Figure 2:**
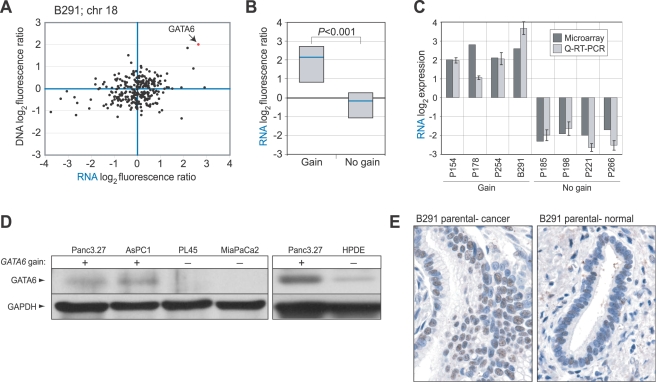
*GATA6* is overexpressed when amplified. (A) Plot of DNA (by array CGH) vs. mRNA (by expression profiling) ratios for genes on chromosome 18 for specimen B291 shows *GATA6* (indicated) to be the most highly expressed gene within the 18q11.2 amplicon. (B) *GATA6* mRNA levels, measured by microarray, are elevated in pancreatobiliary xenografts with compared to without DNA gain at 18q11.2 (*GATA6*). Box plots show 25^th^, 50^th^ and 75^th^ percentiles; *P*-values (Mann-Whitney U-Test) for pairwise comparisons are indicated. (C) Q-RT-PCR validation of microarray-measured GATA6 transcript levels in eight specimens, four each with and without 18q11.2 gain. (D) Western blot analysis of representative pancreatic cancer cell lines indicates *GATA6* (56 kD) is overexpressed at the protein level when amplified; GAPDH serves as a loading control. (E) IHC analysis of GATA6 protein expression (nuclear brown staining) indicates elevated expression in the parent tumor from which xenograft B291 was derived (*left*), in comparison to normal pancreatic duct from the same paraffin section (*right*).

**Figure 3:**
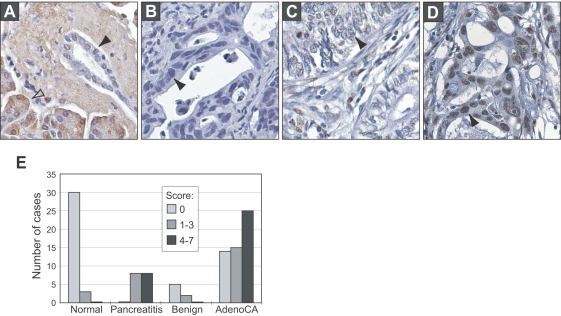
*GATA6* is overexpressed in primary pancreatic tumors. Shown are representative IHC stains for GATA6 protein expression in (A) normal pancreas, and in pancreatic ductal adenocarcinoma with (B) absent, (C) moderate, and (D) strong nuclear staining. Filled arrowheads indicate pancreatic ductal epithelial cells (*A*), or pancreatic adenocarcinoma cells (*B*–*D*). Open arrowhead (*A*) shows non-specific cytoplasmic staining observed in pancreatic acinar cells. (E) Distribution of GATA6 expression among different diagnoses represented on the tissue microarray. The IHC staining score considers both staining intensity and fraction of cells with nuclear staining (see Materials and Methods). GATA6 expression is significantly elevated in pancreatic cancer compared to normal pancreas (*P*<0.001, χ^2^ test).

Consistent with an oncogenic role, *GATA6* exhibited increased mRNA expression by microarray in B291 (Figure 2A) and among the group of xenograft specimens with 18q11.2 gain (Figure 2B; *P* = 0.003, Mann-Whitney U-Test), a finding also confirmed by Q-reverse transcription (RT)-PCR (Figure 2C). In contrast, expression of the neighboring gene *CTAGE1* was not detectable by Q-RT-PCR (data not shown). We also observed increased GATA6 protein levels by Western blot in pancreatic cancer cell lines with 18q11.2 gain, compared to pancreatic cancer cell lines without gain or to the nontumorigenic human pancreatic ductal epithelial line HPDE (Figure 2D), and by immunohistochemistry (IHC) in the parent tumor from which the B291 xenograft was derived (Figure 2E). To assess the frequency with which GATA6 exhibited elevated expression in primary pancreatic cancer, we performed IHC on a tissue microarray (TMA) that included cases of normal pancreas, pancreatitis and pancreatic ductal adenocarcinoma. We observed moderate and strong GATA6 nuclear staining respectively in 15 (28%) and 25 (46%) of 54 primary pancreatic cancers compared to just 3 (9%) and 0 (0%) of 33 normal pancreas specimens surveyed (*P*<0.001, χ^2^ test) (Figure 3). GATA6 expression was also elevated in pancreatitis (Figure 3E). There was no significant relation between GATA6 staining and tumor grade (*P* = 0.18, χ^2^ test).

**Figure 4:**
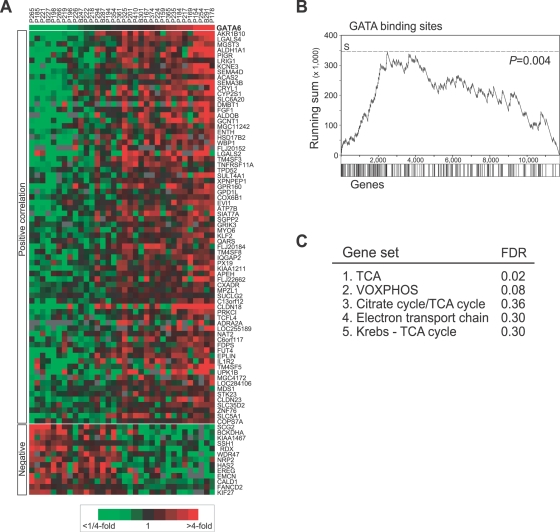
*GATA6* expression signature. (A) Heatmap representation of genes identified by SAM analysis with significantly (FDR<1%) increased (73 genes) or decreased (13 genes) expression in xenografts with GATA6 mRNA levels above the mean. Specimens are ordered by GATA6 expression level; genes are ordered in descending rank of their SAM score. Expression levels are indicated by colorimetric ratio-scale (shown). (B) GSEA identifies enrichment of genes with putative GATA binding sites in xenografts with GATA6 expression levels above the mean. Enrichment is evidenced by the early positive deflection of the Kolmogorov-Smirnov running sum. The significance of the maximum running sum (*S*) was evaluated by comparison to 500 trials with randomly permuted class labels; the *P*-value is the frequency that *S* in the actual data is equaled or exceeded in the permuted data. (C) Top ranking (FDR shown) functional gene sets identified by GSEA to be enriched in xenografts with above-average GATA6 expression levels. TCA: tricarboxylic acid (Krebs) cycle.

Since GATA6 is a transcriptional regulator, we sought to identify co-expressed genes, which might include its downstream transcriptional targets and suggest functional involvements. Using Significance Analysis of Microarrays (SAM) [22], we identified 86 genes whose expression was significantly (False discovery rate, FDR, <1%) increased (73 genes) or decreased (13 genes) in xenografts with elevated GATA6 expression (Figure 4A). The SAM-identified gene set spanned diverse biological processes, and included known cancer genes like *FGF1* and *EVI1*. Gene Set Enrichment Analysis (GSEA) [23] confirmed an enrichment of putative upstream GATA factor binding sites among the genes whose expression correlated with elevated GATA6 levels (*P* = 0.004) (Figure 4B). Interestingly, by GSEA the top functional gene sets associated with elevated GATA6 expression all related to mitochondrial activities connected to oxidative phosphorylation (Figure 4C).

**Figure 5:**
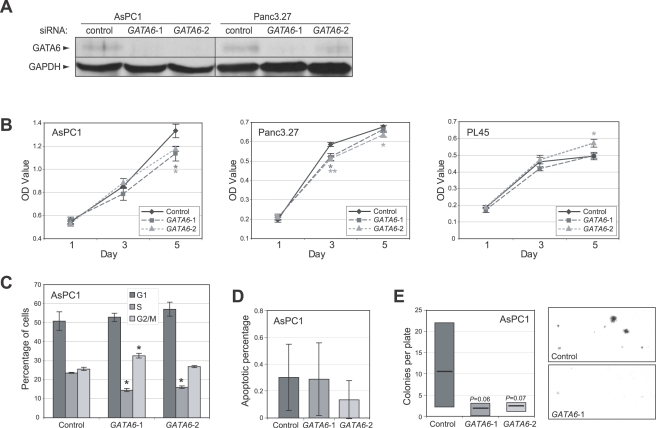
*GATA6* amplification/overexpression contributes to cell proliferation. (A) Confirmation of siRNA-mediated knockdown of GATA6 in AsPC1 and Panc3.27 cells. Two different siRNAs (*GATA6*-1 and *GATA6*-2) were used to target GATA6, along with a non-targeting siRNA pool (control). GATA6 levels assayed by Western blot; GAPDH levels provide a loading control. (B) GATA6 knockdown results in decreased cell proliferation in reduced serum, measured by WST-1 assay, in cells with (AsPC1, Panc3.27) but not without (PL45) *GATA6* gain/overexpression. *, *P*<0.05; **, *P*<0.01 (Student's t-test; *GATA6* compared to control). (C) GATA6 knockdown reduces cell-cycle progression in AsPC1 cells, evidenced by decreased S-phase fraction following BrdU labeling, quantified by flow cytometry. *, *P*<0.05; (Student's t-test; *GATA6* compared to control). (D) GATA6 knockdown does not significantly alter levels of apoptosis, quantified by annexin V staining. (E) GATA6 knockdown reduces colony growth of AsPC1 cells in liquid culture. Box plot illustrates 25^th^, mean and 75^th^ percentile; *P* values (Student's t-test) indicated. Representative fields of Giemsa-stained colonies are shown (*right*).

To directly assess the functional significance of *GATA6* amplification and overexpression in pancreatic cancer, we used RNA interference (RNAi) to target GATA6 knockdown in two pancreatic cancer cell lines, AsPC1 and Panc3.27, with *GATA6* gain and overexpression. Transfection of two independent On-TARGETplus short interfering RNAs (siRNAs) targeting *GATA6*, designed and chemically modified to minimize off-target effects [24],[25], led to decreased GATA6 protein levels (Figure 5A), and to decreased cell proliferation compared to a negative control siRNA pool (Figure 5B). While the reduction in cell proliferation was relatively modest, it was statistically significant and reproducible in multiple independent experiments (not shown). In contrast, siRNA transfection of a pancreatic cancer cell line, PL45, without *GATA6* amplification and overexpression (Figure 2D) did not diminish cell proliferation (Figure 5B, *right* panel), supporting the specificity of *GATA6* targeting. We examined in more detail the effect of GATA6 knockdown in AsPC1 cells, where the reduced cell proliferation was attributable to decreased cell-cycle progression (as evidenced by decreased S-phase fraction; Figure 5C) but not increased apoptosis (Figure 5D). GATA6 knockdown in AsPC1 cells also led to reduced colony formation in liquid culture (Figure 5E).

In complementary experiments, we attempted to overexpress GATA6 by retroviral transduction in nontumorigenic human pancreatic ductal epithelial HPDE cells, and in the pancreatic cancer cell line PL45 harboring activated KRAS but no 18q11.2 gain. Though GATA6 expression was initially detected in infected cells by Western blot (data not shown), expression was lost upon expansion of cell pools under selection, suggesting GATA6 conferred negative fitness in these cell contexts.

## Discussion

A main objective of our study was to comprehensively catalog CNAs in pancreatobiliary cancers. Genomic profiling of 31 pancreatic and 6 distal bile duct cancer xenografts identified numerous focal high-level DNA amplifications and homozygous deletions, thereby pinpointing known and candidate cancer genes. Known cancer genes included focal amplifications of *MYC* (8q24.21), *KRAS* (12p12.1) and *AKT2* (19q13.2), and homozygous deletions of *TGFBR2* (3p24.1) and *CDKN2A* (9p21.3). Other focal changes suggest entirely new pathobiology. For example, homozygous deletion of *TLR3* (Toll-like receptor 3) (4q35.1), which functions in the innate immune response and is also highly expressed in pancreas [26],[27], suggests a possible role of infection in pancreatic carcinogenesis.

Prominent among novel oncogene candidates we identified *GATA6* amplification at 18q11.2. GATA6 is one of six members of the mammalian GATA family of transcriptional regulators, each having two zinc finger domains and binding the common DNA sequence element (A/T)GATA(A/G) [19]. GATA factors 1–3 are expressed mainly in hematopoietic lineages, while GATA factors 4–6 are expressed in various tissues derived from mesoderm and endoderm, including the heart, liver, lung, gut, ovary and testis, where they function in cell lineage specification [19],[28]. In relation to pancreas development in the mouse, GATA4 and GATA6 are expressed in both endocrine and exocrine cell precursors, while in the adult pancreas expression of GATA4 and GATA6 is restricted to the exocrine and endocrine compartment, respectively [20],[29]. Recently, GATA4 and GATA6 have both been shown to be required for normal pancreas specification and development [20],[21]. While *GATA6* has been linked to pancreas development, our findings now also connect *GATA6* to pancreatic cancer, where *GATA6* amplification and resultant overexpression contribute significantly (albeit at modest levels) to oncogenic phenotypes (cell proliferation, cell-cycle progression and colony formation) of pancreatic cancer cells.

Given its connection to development and cell specification, an oncogenic role of *GATA6* might seem surprising. Indeed, *GATA6* has been characterized as a TSG in other cell contexts [30],[31], and inactivating mutations have been identified in human malignant astrocytomas [31]. Nonetheless, other cell lineage-specific transcription factors have been found amplified in cancers, including *MITF* in melanoma [32], *AR* in hormone-independent prostate cancer [33], *ESR1* in breast cancer [34], and most recently *NKX2-1* (*TITF*) in lung cancer [17], [35]–[37]. The altered expression of such transcriptional regulators, having normal roles in lineage proliferation or survival, might be needed for tumor survival and progression in some cellular and genetic contexts, indicating a state of “lineage-dependency” [38]. More generally, the deregulated expression of transcription factors with roles in normal development reflects the principle of “oncology recapitulating ontogeny” [39]. While we detected *GATA6* amplification primarily in pancreatobiliary cancers, GATA6 expression is not restricted to the developing pancreas, and therefore it remains to be determined whether GATA6 might have an oncogenic role in other cell lineages.

Another characteristic of lineage-specific oncogenes is that their oncogenic activity appears to be highly cell and genetic context dependent. MITF expression is growth inhibitory in normal human melanocytes [40], but in the context of BRAF activation (along with TP53 and RB1 pathway inactivation) leads to growth factor and anchorage independent growth [32]. Likewise, TITF1 is growth inhibitory when expressed in immortalized human lung epithelial cells [35], but promotes cell proliferation and survival when amplified in lung cancers [17],[37]. Consistent with these findings, GATA6 expression imparted negative fitness in immortalized human pancreatic ductal epithelial cells (HPDE), and in a pancreatic cancer cell line (PL45) with KRAS activation but no 18q11.2 gain. Additional studies are needed to clarify the genetic context of GATA6 oncogenic function.

While *GATA6* was amplified in 19% of xenograft specimens, it was highly expressed at the protein level in 46% of primary pancreatic tumors surveyed. This finding suggests that GATA6 expression is likely elevated by mechanisms other than gene amplification in a substantial subset of cases. We also noted increased GATA6 expression in pancreatitis, which is a known risk factor for developing pancreatic cancer [41], and suggests a possible mechanistic link. As noted above, GATA4 is also expressed during normal pancreas development, though unlike GATA6 its expression is retained in the adult exocrine pancreas. Of interest, we have also observed DNA gains spanning *GATA4* at 8p23.1 in a subset of xenografts (not shown), though none having focal DNA amplification. Additional studies are needed to examine the function, if any, of GATA4 in pancreatobiliary cancer.

Our analysis of gene-expression patterns correlated with GATA6 transcript levels has revealed an intriguing association with genes functioning in mitochondrial oxidative phosphorylation. Such altered mitochondrial activity might contribute to carcinogenesis through changes in cell metabolism, reactive oxygen species (ROS) production, or (through altered mitochondrial membrane potential) mitochondrial-associated apoptotic pathways [42],[43]. In the latter regard, it is notable in that we did not observe an effect of GATA6 knockdown on apoptosis, but rather on cell-cycle progression. While the exact connection to mitochondrial oxidative phosphorylation remains to be elucidated, it is of interest that a similar “OxPhos” expression pattern was recently identified in a subset of diffuse large B-cell lymphomas [44].

Since GATA6 is expressed in normal adult tissues like the endocrine pancreas, the lung, liver and heart [18], it is itself unlikely to become a useful target for therapy. However, future investigations will more precisely define the transcriptional effectors and pathways through which GATA6 mediates its oncogenic function, some of which might become important molecular targets. In conclusion, our genomic profiling and functional studies define *GATA6* as a candidate lineage-specific oncogene in pancreatobiliary cancers, a finding which should lead to new opportunities for therapeutic intervention.

## Materials and Methods

### Specimens

Pancreatic and distal bile duct cancer xenografts were generated as described [14] at the Johns Hopkins Hospital, with Institutional Review Board (IRB) and Institutional Animal Care and Use Committee approval. Briefly, a 1 mm^3^ piece of the primary tumor was soaked in Matrigel (Collaborative Biomedical Research), then implanted subcutaneously in a nu/nu mouse. Engrafted tumors were harvested when they reached 1–2 cm in diameter. Tumor cell enrichment was confirmed by H&E-stained frozen section. From adjacent pieces of the xenograft, DNA was isolated using the Qiagen DNeasy Tissue kit, and RNA using the Trizol (Invitrogen) method. Pancreatic cancer cell lines were obtained from the American Type Culture Collection, and the HPDE cell line [45] was kindly provided by Dr. Ming Tsao (University of Toronto).

### Array CGH and Expression Profiling

cDNA microarrays were obtained from the Stanford Functional Genomics Facility and included 39,632 human cDNAs, representing 22,279 mapped human genes (18,049 UniGene clusters [46], together with 4,230 additional mapped ESTs not assigned UniGene IDs). Array CGH and expression profiling were performed according to our published protocols [47],[48]. For array CGH, 4 µg of genomic DNA from each test sample was random-primer labeled with Cy5 and co-hybridized to the microarray along with 4 µg of Cy3-labeled sex-matched normal leukocyte reference DNA from a single donor. For gene-expression profiling, 50 µg of total RNA from each sample and 50 µg of “universal” reference RNA (derived from 11 different established human cell lines) were differentially labeled with Cy5 and Cy3, respectively, and co-hybridized to cDNA microarrays. Following overnight hybridization and washing, arrays were imaged using a GenePix 4000B scanner (Molecular Devices). Fluorescence ratios were extracted using SpotReader software (Niles Scientific), and the data uploaded into the Stanford Microarray Database (SMD) [49] for storage, retrieval and analysis. The complete microarray datasets are available at SMD and at the Gene Expression Omnibus (GEO) (accession GSE11152).

### Microarray Data Analysis

Background-subtracted fluorescence ratios were normalized by mean centering genes for each array. For array CGH analysis, we included for subsequent analysis only well-measured genes with Cy3 reference-channel fluorescence signal intensity at least 1.4-fold above background in at least 50% of samples. Map positions for arrayed cDNA clones were assigned using the NCBI genome assembly, accessed through the UCSC genome browser database (NCBI Build 36). For genes represented by multiple arrayed cDNAs, the average fluorescence ratio was used. DNA gains and losses were identified by the fused lasso method [50]. We defined high-level DNA amplifications and presumptive homozygous deletions as contiguous regions identified by fused lasso with at least 50% of genes displaying fluorescence ratios ≥3 or ≤0.25, respectively. For expression profiling, fluorescence ratios were normalized for each array, and then well-measured genes (fluorescence intensities for the Cy5 or Cy3 channel at least 1.5-fold above background) were subsequently “mean-centered” (i.e. reported for each gene relative to the mean ratio across all samples).

SAM analysis [22] was performed using the 2-class method, comparing xenograft specimens with above and below mean GATA6 mRNA levels. GSEA [23] was carried out as described [51]. Genes with putative GATA binding sites (within the first 1-Kb upstream promoter sequence) were defined using MATCH software ([52]; default settings set to minimize false positives), applied to the common binding site matrix V$GATA_Q6 (all six GATA factors share a common DNA binding site, (A/T)GATA(A/G) [19]). To assess enrichment of GATA binding sites, the absolute value of the GSEA metric (Pearson correlation) was used in order to consider both upregulated and downregulated targets. GSEA using 522 functional gene sets was carried out as described [23].

### High-Definition 18q11.12 CGH Microarray

A custom ultra high-definition CGH microarray was designed and obtained from Agilent Technologies [53]. The array included 4,362 probes tiling 1.5 Mb (Mb 17.5–19.0) of 18q11.2 with an average inter-probe spacing of 344 nt, and an additional 23,652 probes spanning the remaining genome for data normalization. DNAs were labeled as above, then hybridized to the Agilent array following the manufacturer's instructions, except using a 40 hr hybridization time (rather than the recommended 24 hr). Arrays were scanned using an Agilent G2505B scanner, and data extracted and normalized using Agilent Feature Extraction software (version 9.1) with default settings.

### PCR Assays

To validate homozygous deletions (see Table 1), we used gene-specific primer-pairs to PCR amplify genomic DNA from xenograft specimens. Primer-pairs for genes flanking the regions of homozygous deletion, and designed to have a distinguishable fragment size, were included in the PCR reactions as internal controls, and normal DNA was used as positive control for primer pairs. PCR was performed on an Applied Biosystems GeneAmp 9700, using 40 ng DNA template, 1× PCR buffer (Applied Biosystems), 200 µM dNTPs, 2.0 mM MgCl_2_, 10 pmol each individual primer (Table S1), and 1 U of AmpliTaq Gold DNA polymerase (Applied Biosystems) in a 10 µl reaction. The reaction conditions were: 95°C 10 min initial denaturation, followed by 35 cycles (94°C 30 s; annealing temp (see Table S1) 30 s; 72°C 30 s), and a final extension of 72°C 7 min. PCR products were resolved by gel electrophoresis on a 1.8% TAE agarose gel, and visualized using a UVP gel documentation system.

To validate *GATA6* amplification in B291, we carried out Q-PCR using the Quantitect SYBR Green PCR kit (Qiagen) on an ABI 7500 sequence detection system as per manufacturer's instructions. PCR was initiated at 95°C for 15 min (to activate the modified Taq polymerase), followed by a 40 cycle amplification (95°C 15 s, 58°C 30 s, 72°C 30 s). Melting curve analysis was performed to ensure specific PCR product while excluding primer dimers. We used the comparative CT method [54] to calculate relative DNA levels normalized to *NPC1* (a gene located outside the 18q11.2 amplicon and not exhibiting CNA), which we then expressed as a ratio to the Ct value of *GATA6* (also normalized to *NPC1*) obtained from normal DNA. PCR primer sequences are listed in Table S1.

To validate microarray-measured GATA6 mRNA levels, we carried out Q-RT-PCR using the QuantiTect SYBR Green RT-PCR Kit according to the manufacturer's instructions. The reaction mixture was first incubated at 50°C for 30 min for reverse transcription, then Q-PCR was carried out as above. For each specimen, relative levels of GATA6 transcript were calculated as the ratio of Ct value of GATA6 to that of GAPDH. Ratio values were then converted to log_2_ scale and normalized to the mean across all specimens. PCR primer sequences are listed in Table S1.

### FISH

Probe labeling and FISH were performed using Vysis reagents according to the manufacturer's protocols. A locus-specific BAC mapping to *GATA6* at 18q11.2 (RP11-1083G24; BACPAC Resources Centre) was labeled with SpectrumOrange, and co-hybridized with SpectrumGreen-labeled chromosome 18 centromere probe (CEP18; Vysis). Chromosomal locations of BACs were validated using normal metaphase slides (not shown). Slides were counterstained with DAPI, and imaged using an Olympus BX51 fluorescence microscope with Applied Imaging Cytovision 3.0 software.

### Immunohistochemistry

A tissue microarray (TMA) was constructed using a tissue arrayer (Beecher Instruments) and archived formalin-fixed, paraffin-embedded pancreatic tissue specimens from Stanford University, with IRB approval. The TMA contained 1.2 mm cores representing normal pancreas (33 cases), pancreatitis (16), other benign diagnoses (9), and pancreatic ductal adenocarcinoma (54). For immunohistochemistry, a 4 µm section was cut from the tissue microarray block, de-paraffinized in Citrisolv (Fisher Scientific), and hydrated in a graded series of alcohol solutions. Heat-induced antigen retrieval was performed by microwave pretreatment in citrate (1 mM, pH 6.0) for 15 minutes before staining. Endogenous peroxidase was blocked by preincubation with 1% hydrogen peroxide in phosphate-buffered saline. A GATA6 mouse monoclonal antibody (R&D systems) was used at 1∶10 dilution for 30 min. Chromogenic detection was carried out using a peroxidase-conjugated secondary antibody and DAB reagents provided with the Envision detection kit (Dako). Nuclear staining intensity (absent, weak, medium, strong; 0–3 scale) and fractional epithelium staining (up to 25%, 50%, 75%, 100%; 1–4 scale) were each recorded, then summed for a final staining score.

### siRNA Transfections

On-TARGETplus siRNAs targeting *GATA6*, along with a negative control siRNA pool (ON-TARGETplus siCONTROL Non-targeting Pool), were obtained from Dharmacon. Sequences of siRNAs are listed in Table S1. Cell lines were maintained at 37°C in complete media of RPMI-1640 (Invitrogen), 10% FBS, 50 U/ml penicillin, and 50 U/ml streptomycin. For transfection, 100,000–250,000 cells were seeded per 6-well plate well, and transfected using Lipofectamine 2000 reagent (Invitrogen) according to the manufacturer's protocol. Cells were transfected with a final concentration of 50 nM siRNA for 6 hrs, subsequently replaced with reduced serum (2% FBS) growth media, where siRNA-mediated phenotypic effects were more reproducibly observed.

### Western Blot

72 hours post-transfection, cells were lysed in 1× RIPA Lysis buffer (Upstate/Chemicon) supplemented with 1× Complete Protease Inhibitor (Roche, Indianapolis, IN), 0.1 mM sodium orthovanadate, 1 mM sodium fluoride and 1 mM PMSF, and protein quantified using the DC Protein Assay (Biorad). For Western blot, 25–40 µg protein lysate was electrophoresed on a 10% Tris/glycine polyacrylamide gradient gel (Biorad) and transferred to PVDF membrane (Biorad). After blocking in TBS-T buffer (20 mM Tris-HCl pH 7.4, 0.15 M NaCl, 0.1% Tween 20) with 5% dry milk for 30 min, blots were incubated sequentially with primary antibody at 4°C overnight, then HRP-conjugated secondary antibody at room temp for 45 min. Antibodies were used as follows: anti-GATA6 rabbit polyclonal antibody (1∶200; Santa Cruz Biotechnology); anti-GAPDH rabbit polyclonal antibody (1∶5,000 for loading control; Santa Cruz Biotechnology); HRP-conjugated anti-rabbit IgG (1∶20,000, Pierce). Detection was carried out using the ECL kit (Amersham Biosciences).

### Cell Proliferation Assay

24, 72 and 96 hours post-transfection, cell proliferation was quantified by colorimetry based on the metabolic cleavage of the tetrazolium salt WST-1 in viable cells, according to the manufacturer's protocol (Roche). WST-1 reagent was added at 1/10^th^ the culture volume and incubated at 37°C for 30 min. Absorbance was then measured at 450 nm with reference to 650 nm using a Spectra Max 190 plate reader (Molecular Devices). Transfections were performed in triplicate and average (±1 SD) OD reported.

### Cell-Cycle Analysis

72 hours post-transfection, cell-cycle distribution analysis was performed by flow cytometry using the BrdU-FITC Flow kit (BD Biosciences) per the manufacturer's instructions. Cells were incubated with 10 µM BrdU at 37°C for 4 hrs, then fixed and permeabilized with Cytofix/Cytoperm buffer (BD Biosciences). Cellular DNA was treated with DNase at 37° C for 1hr to expose incorporated BrdU, then cells were stained with anti-BrdU FITC antibody (to quantify incorporated BrdU) and 7-aminoactinomycin D (7-AAD; to quantify total DNA content). 10,000 events were scored by FACSCalibur (BD Biosciences) and analyzed using CellQuest software (BD Biosciences). Transfections were performed in triplicate and average (±1 SD) cell-cycle fractions reported.

### Liquid Colony Formation Assay

24 hours post-transfection, 200 cells were plated each onto ten 10 cm dishes in complete media. After 2 weeks, surviving cells were stained with Giemsa (Sigma-Aldrich) for 15 min, and visible colonies counted on a light box.

### Apoptosis Assay

72 hours post transfection, apoptosis was assayed by annexin V staining, quantified by flow cytometry using the Vybrant Apoptosis Assay kit (Invitrogen) as per the manufacturer's instructions. Floating cells and trypsinized adherent cells were pooled and resuspended in 200 µl annexin binding buffer. 2.5 µl Alexa Fluor 488 annexin V and 1 µl of 100 µg/ml propidium iodide (PI) solution were added and cells incubated for 15 min at room temp. Cells were then resuspended in equal volume of annexin binding buffer and analyzed immediately by flow cytometry. 10,000 events were scored by FACSCalibur and analyzed using CellQuest software. Transfections were performed in triplicate, and average (±1 SD) percent apoptosis reported.

## Supporting Information

Figure S1 Representative PCR-validations of homozygous deletion. (A) 3p24.1 deletion. TGFBR2, located within the presumptive homozygous deletion, is PCR-amplified from normal genomic DNA, but not from pancreatic cancer xenograft P224. (B) 9p21.2 deletion. MOBKL2B, within the deletion, is PCR-amplified from normal genomic DNA but not from P201.
(0.22 MB PDF)

Table S1 Primer/siRNA sequences.
(0.02 MB XLS)
