# Supplementary material for: Genomic Profiling Identifies GATA6 as a Candidate Oncogene Amplified in Pancreatobiliary Cancer
Source: PLoS Genet. 2008 May 23;4(5):e1000081. doi: 10.1371/journal.pgen.1000081 (PMC2413204; doi:10.1371/journal.pgen.1000081)
Supplement: Figure S1 — Representative PCR-validations of homozygous deletion. (A) 3p24.1 deletion. TGFBR2, located within the presumptive homozygous deletion, is PCR-amplified from normal genomic DNA, but not from pancreatic cancer xenograft P224. (B) 9p21.2 deletion. MOBKL2B, within the deletion, is PCR-amplified from normal genomic DNA but not from P201. (0.22 MB PDF) [file pgen.1000081.s001.pdf]

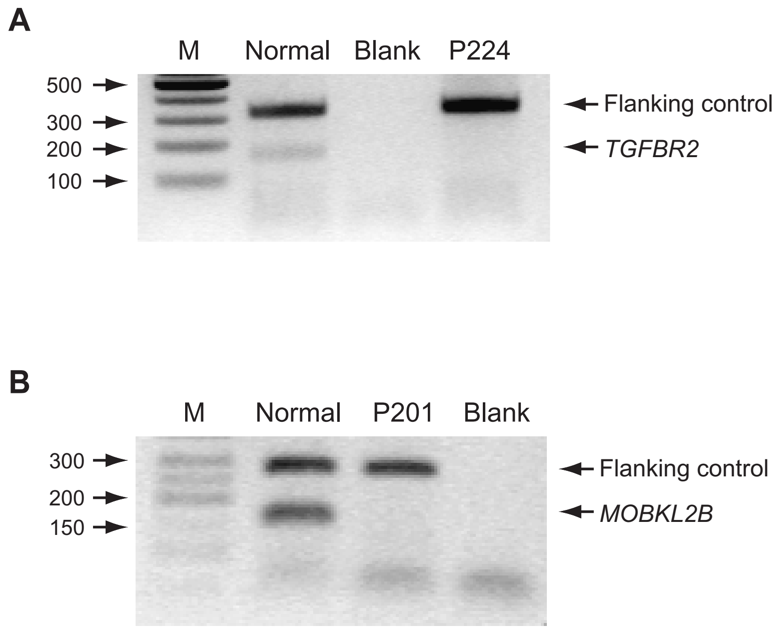

Figure S1. Representative PCR-validations of homozygous deletion. **(A)** 3p24.1 deletion. *TGFBR2*, located within the presumptive homozygous deletion, is PCR-amplified from normal genomic DNA, but not from pancreatic cancer xenograft P224. **(B)** 9p21.2 deletion. *MOBKL2B*, within the deletion, is PCR-amplified from normal genomic DNA but not from P201.
